# Prevalence of cancer-related fatigue, associated factors and adult cancer patients’ experiences at Hawassa University Comprehensive Specialized Hospital in Ethiopia: a mixed methods study

**DOI:** 10.3389/fonc.2024.1480246

**Published:** 2024-11-01

**Authors:** Tseganesh Asefa, Gedamnesh Bitew, Hiwot Tezera, Winta Tesfaye

**Affiliations:** ^1^ Department of Medical Nursing, School of Nursing, College of Medicine and Health Science, University of Gondar, Gondar, Ethiopia; ^2^ Department of Epidemiology and Biostatics, School of Medicine, College of Medicine and Health Science, Injibara University, Injibara, Ethiopia; ^3^ Department of Biochemistry, School of Medicine, College of Medicine and Health Science, University of Gondar, Gondar, Ethiopia; ^4^ Department of Physiology, School of Medicine, College of Medicine and Health Science, University of Gondar, Gondar, Ethiopia

**Keywords:** cancer-related fatigue, cancer patients, brief fatigue inventory, experience, Ethiopia, Hawassa

## Abstract

**Purpose:**

Cancer-related fatigue is a prevalent issue affecting 50–90% of cancer patients who experience fatigue at diagnosis, during therapy, and often for months or years after the completion of therapy. This study aimed to explore the prevalence of cancer-related fatigue, associated factors, and adult cancer patients’ experiences at Hawassa University Comprehensive Specialized Hospital in Ethiopia.

**Methods:**

A mixed-method study was conducted from February 25 to May 15, 2023, via cross-sectional descriptive and phenomenological approaches. The validated Amharic Brief Fatigue Inventory scale and semistructured interview guide were used. The data were processed via Epi-data version 4.4.3.1 and SPSS version 24, with logistic regression analysis. The interview records and field notes were transcribed and translated from Amharic to English and then analysed thematically.

**Results:**

All participants (100%) completed the study, with 77.4% reporting significant fatigue. Fatigue was strongly associated with uninsured medical expenses (P = 0.008, OR = 3.22), late-stage cancer (P = 0.000, OR = 6.11), anaemia (P = 0.009, OR = 3.71), and comorbidities (P = 0.000, OR = 7.22). From the in-depth interviews with 16 participants, two main themes emerged: financial strain (giving up basics, and inability to work) and disease progression (intensified symptoms, increased treatment side effects, and managing multiple conditions).

**Conclusion:**

This study revealed that 77.4% of cancer patients experience significant fatigue, which is linked to a lack of medical insurance, late-stage cancer, anaemia, and comorbid conditions. Financial strain limits access to care, whereas disease progression and managing multiple conditions intensify fatigue. Early intervention, financial support, and integrated care are crucial for reducing fatigue and improving quality of life. Future research should focus on multicentre and longitudinal studies to improve generalizability and track fatigue progression over time.

## Introduction

Cancer incidence and mortality are expected to affect 18.1 million and 9.6 million people worldwide, respectively. Each year, Central and Southern America, as well as Africa, account for more than 60% of all cancer cases (1, 2). Similarly, 506,000 deaths and 752,000 new cases were recorded in Sub-Saharan Africa alone. There is still a growing public health impact in Sub-Saharan Africa as a whole. Every year, Ethiopia has more than 150,000 new cases of cancer, accounting for 4% of all cancer-related deaths (3, 4).

Fatigue is described by the National Comprehensive Cancer Network (NCCN) as a continuous, subjective feeling of exhaustion associated with cancer or cancer therapy that prevents one from performing daily tasks. It is a multifaceted, complex symptom that causes physical pain, distress, and functional limitations, and it reflects a person’s likelihood of surviving the disease and maintaining a high-quality life (5–7). Individuals undergoing cancer treatment, such as chemotherapy and radiation, are reportedly more likely to experience fatigue, with a range of 50–90%. Owing to these factors, fatigued individuals have a significant emotional impact on their quality of life and happiness, in addition to affecting their social roles in their family and community (8–11).

The occurrence or exacerbation of fatigue in individuals with cancer is associated with factors such as sex, age, employment status, independent living, performance status, cancer type and stage, infections, comorbidities, anaemia, and the use of antiemetic drugs or cancer therapies (12–14).

Cancer-related fatigue is an important and often neglected aspect of cancer care, particularly in low-income countries. Understanding the prevalence of fatigue and its associated factors is crucial for improving care and treatment outcomes, as highlighted in previous studies, given that fatigue significantly affects quality of life. However, there is limited qualitative insight into individuals’ personal experiences with fatigue. A mixed-methods approach can address issues that may not be fully captured in either quantitative or qualitative studies. Additionally, the triangulation of study results enhances the ability to combine statistical data with the detailed experiences of patients. This ultimately leads to a comprehensive understanding of the issue, enabling effective interventions and improved patient care. This mixed-method design study quantifies the prevalence and identifies associated factors while further exploring the depth of the subjective experience of adults with cancer at Hawassa University Comprehensive Specialized Hospital.

## Methods and materials

### Study design, period, and setting

A mixed-method study employing both a cross-sectional descriptive and phenomenological approach was conducted from February 25 to May 15, 2023, at the cancer treatment centre of Hawassa University Comprehensive Specialized Hospital (HUCSH) in Ethiopia. This is the only comprehensive specialized hospital that provides cancer treatment and management services for more than 18 million people living in Sidama, the Southern Nations and Nationalities, and some Oromia regions of Ethiopia. The cancer treatment centre in the facility provides services for more than 1200 individuals with cancer annually and has two subunits: adult and infant oncology.

### Study population

All adults aged 18 years and older with a confirmed diagnosis of cancer who received chemotherapy at HUCSH during the data collection period were included in the study. Individuals were excluded if they had cognitive impairments, or encountered language barriers that hindered their understanding of the study’s purpose.

### Sample size determination and sampling technique

#### For the quantitative study

The study sample size was determined via the single population proportion formula. $\text{n}=\frac{\left(Z\alpha /2\right)2\;p\;\left(1-p\right)\;}{d2}$ , the proportion of fatigue among individuals with cancer is 77.3% (15), assuming a 95% confidence level and a 5% margin of error as follows:


$$\text{n}=\frac{((1.96{)}^{2}*\;0.773*\;\left(1-0.773)\right)}{(0.05{)}^{2}}=270$$

where n= minimum sample size, P= estimated proportion of cancer-related fatigue (p= 0.773), d = the margin of sampling error tolerated (5%), and Zα/2 = the standard normal distribution at 95% CI =1.96. After adding a 10% nonresponse rate, the final sample size was 297.

A systematic random sampling technique was used to approach the study participants at the cancer treatment centre. The sampling interval was calculated via the formula $\text{k}=\frac{Nt}{n}$ .

where Nt= the estimated total population size at one typical month from the hospital record (600), n= the total sample size, and k =600/270 = 2.22 ≈ 2.

The first participant was selected at random via a lottery, and then additional participants were chosen every two individuals until the desired sample size was reached.

#### For the qualitative study

A non-probability heterogeneous purposive sampling approach was used to ensure the collection of adequate information for the study. Data saturation was reached during the 13th interview, and three additional interviews were conducted to confirm that no new themes or information emerged, resulting in a total of 16 interviews.

### Operational definition

Fatigue: Respondents who score on a brief fatigue inventory ≥ 1 (16).

No fatigue: Respondents who score a brief fatigue inventory of 0 (16).

Late stage: The term “late stage at presentation” refers to cases diagnosed at FIGO stages III and IV (17).

Anaemia: A haemoglobin level less than 12.0 g/dl and classified as anaemic (18).

Comorbidity: Comorbidity data were obtained from medical records via the Carlson comorbidity index. The presence of any conditions mentioned in the Carlson comorbidity index (19) other than cancer at diagnosis, which was designated “yes” in the checklist.

### Data collection tools and procedures

#### For the quantitative study

The eligible study participants were approached, and they provided informed written consent regarding their willingness to participate in the study. An interview followed. The information was gathered via a structured interviewer-administered questionnaire and a chart review technique. The questionnaire has three sections—sociodemographic characteristics, clinical characteristics, and a validated Brief Fatigue Inventory (BFI) measure cancer-related fatigue (CRF). The first three questions assess the severity of fatigue as the worst fatigue, usual fatigue, and fatigue now and during the past 24 hours, with each item rated from 0 (no fatigue) to 10 (fatigue as bad as you can imagine). For analysis purposes, ‘0’ was considered no fatigue, and ≥ ‘1’ was considered fatigue (16).

After the completion of the interview, the client’s chart was also reviewed to retrieve some clinical and treatment-related information. Three trained BSc nurses were recruited as data collectors, and a nurse with an MSc degree supervised the data collection process.

#### For the qualitative study

An interview guide was utilized to collect qualitative data and acquire a better understanding of how individuals value the impact of fatigue on their life. The principal investigator developed this guide in English on the basis of prior studies, which was then translated into Amharic by a fluent speaker of both languages. The principal investigator conducted interviews that lasted an average of 34 minutes. During the interviews, the investigator used a recorder to take field notes. All the documents were securely stored, and the digital version of the material was password protected and accessible only by the researcher.

### Data quality control

#### For the quantitative study

To ensure the quality of the data, the recruited data collectors underwent training sessions lasting half a day. They were trained on the objective, confidentiality of information, relevance of the study, respondents’ rights, pretest, informed consent, and interview techniques. Next, the questionnaire was pretested on 5% of the sample in the oncology unit at the University of Gondar Comprehensive Specialized Hospital to ensure consistency of the survey tool, and modifications were made accordingly. Close supervision was undertaken during the data collection. Furthermore, the tool was tested for internal consistency (reliability) and a Cronbach’s alpha of α = 0.81 was obtained.

#### For the qualitative study

The data quality of qualitative studies is ensured through adherence to principles of transferability, credibility, confirmability, and dependability (20, 21).

Transferability refers to ‘the extent to which the findings can be transferred to other settings or groups’ (22). In qualitative research, the intention is not to generalize the findings (23). Instead, this study used qualitative data to triangulate or validate results from the quantitative study. Transferability was achieved by collecting detailed life experiences from a subsample of cancer patients. Credibility was maintained through open communication with participants to verify the authenticity of their experiences. Confirmability was ensured by supporting findings with quotations, and dependability was established by providing detailed information about data collection, timing, location, and analysis.

### Data analysis

#### For the quantitative study

After the completeness and consistency of the collected data were checked, the data were coded and entered into Epi-data version 4.4.3.1 and then exported to SPSS version 25 for analysis. During the process of analysis, the frequency distributions and percentages of different variables were computed to describe and summarize the basic sociodemographic characteristics of the respondents. Bivariate and multivariate analyses were used to examine the associations between the dependent and independent variables. The level of significance was determined by the 95% confidence interval and a P value less than 0.05 for associations.

#### For the qualitative study

The data from individual interview records and field notes were also transcribed and translated from Amharic to English verbatim by a fluent speaker of both languages. The researcher entered and saved the data as Microsoft Word files. The codes were first organized into subthemes, which were then grouped to develop the main themes. The analysis was conducted using thematic analysis techniques.

## Results

### Quantitative study results

#### Sociodemographic characteristics of the participants in the quantitative study

Among the 297 participants, more than half (55.6%) were male, and less than half (46.1%) were aged 40–60 years, representing a significant portion of middle-aged participants. Notably, 36.7% were aged 18-39 years, representing a considerable portion of the younger participants. Approximately 154 (51.9%) of the participants were urban residents. More than half (56.6%) of the participants were married. One-third (33.0%) of the participants did not attend formal education, and 34.7% reported a monthly household income of less than 500 birrs. However, approximately one hundred forty-three (48.1%) of the participants had medical insurance (**Table 1**).

**Table 1:** Sociodemographic characteristics of the participants in the quantitative study (n = 297).

Variable	Category	Frequency	Percent %
Age	18-39	109	36.7%
	40-60	137	46.1%
	>60	51	17.2%
Gender	Male	165	55.6%
	Female	132	44.4%
Residence	Urban	154	51.9%
	Rural	143	48.1%
Marital status	Single	40	13.5%
	Married	168	56.6%
	Divorced	47	15.8%
	Widowed	42	14.1%
Level of education	No formal education	98	33.0%
	Primary school	66	22.2%
	Secondary school	68	22.9%
	Above secondary school	65	21.9%
Occupation status	Governmental	35	11.8%
	Private	15	5.1%
	Merchant	45	15.2%
	Student	15	5.1%
	Farmer	116	39.1%
	House wife	44	14.8%
	Retire	22	7.4%
	Others	5	1.7%
Household income per capital	<500 birr/month	103	34.7%
	500-1000 birr/month	78	26.3%
	1100-2000 birr/month	55	18.5%
	>2000 birr/month	61	20.5%
Medical insurance	Insured	143	48.1%
	Uninsured	154	51.9%

#### Clinical characteristics of the participants in the quantitative study

Among the two hundred ninety-seven participants, approximately one-third (31.6%) were admitted to the inpatient unit. Two hundred thirteen (71.7%) of the participants had stage III or IV disease. Nearly half (53.5%) of the participants had no sign of infection. Approximately three-quarters (74.4%) of the participants had local and metastatic disease. More than three-quarters (77.7%) were receiving chemotherapy. Half of the participants (50.5% and 49.5%, respectively) reported pain and fatigue status to their physicians. Two-thirds (66.7%) of the participants used antiemetic drugs, whereas more than half (54.2%) of the participants had comorbid diseases (**Table 2**).

**Table 2:** Clinical characteristics of the participants in the quantitative study (n = 297).

Variable	Category	Frequency	Percentage
Type of admission	Out patient	203	68.4%
	In patient	94	31.6%
Stage of cancer	Stage I	55	18.5%
	Stage II	29	9.8%
	Stage III	82	27.6%
	Stage IV	131	44.1%
Presence of infection	Yes	138	46.5%
	No	159	53.5%
Diseases status	No evidence	76	25.6%
	Local	93	31.3%
	Metastasis	128	43.1%
Chemotherapy cycle	1^st^ cycle	43	14.5%
	2^nd^ cycle	38	12.8%
	3^rd^ cycle	38	12.8%
	4^th^ cycle	82	27.6%
	5^th^ cycle and above cycle	96	32.3%
Type of cancer treatment	Chemo & Radiation	113	38.0%
	Chemotherapy	118	39.7%
	Surgery	66	22.2%
Use of anti-pain	Yes	150	50.5%
	No	147	49.5%
Use of antiemetic	Yes	198	66.7%
	No	99	33.3%
Reporting to physician	Yes	147	49.5%
	No	150	50.5%
Anaemic status	Yes	170	57.2%
	No	127	42.8%
Comorbidity diseases	Yes	161	54.2%
	No	136	45.8%
Type of comorbidity diseases	Hypertension	27	9.1%
	HIV/ADIS	28	9.4%
	DM	16	5.4%
	Others	20	6.7%

#### The prevalence of fatigue among the study participants

**Figure 1:**
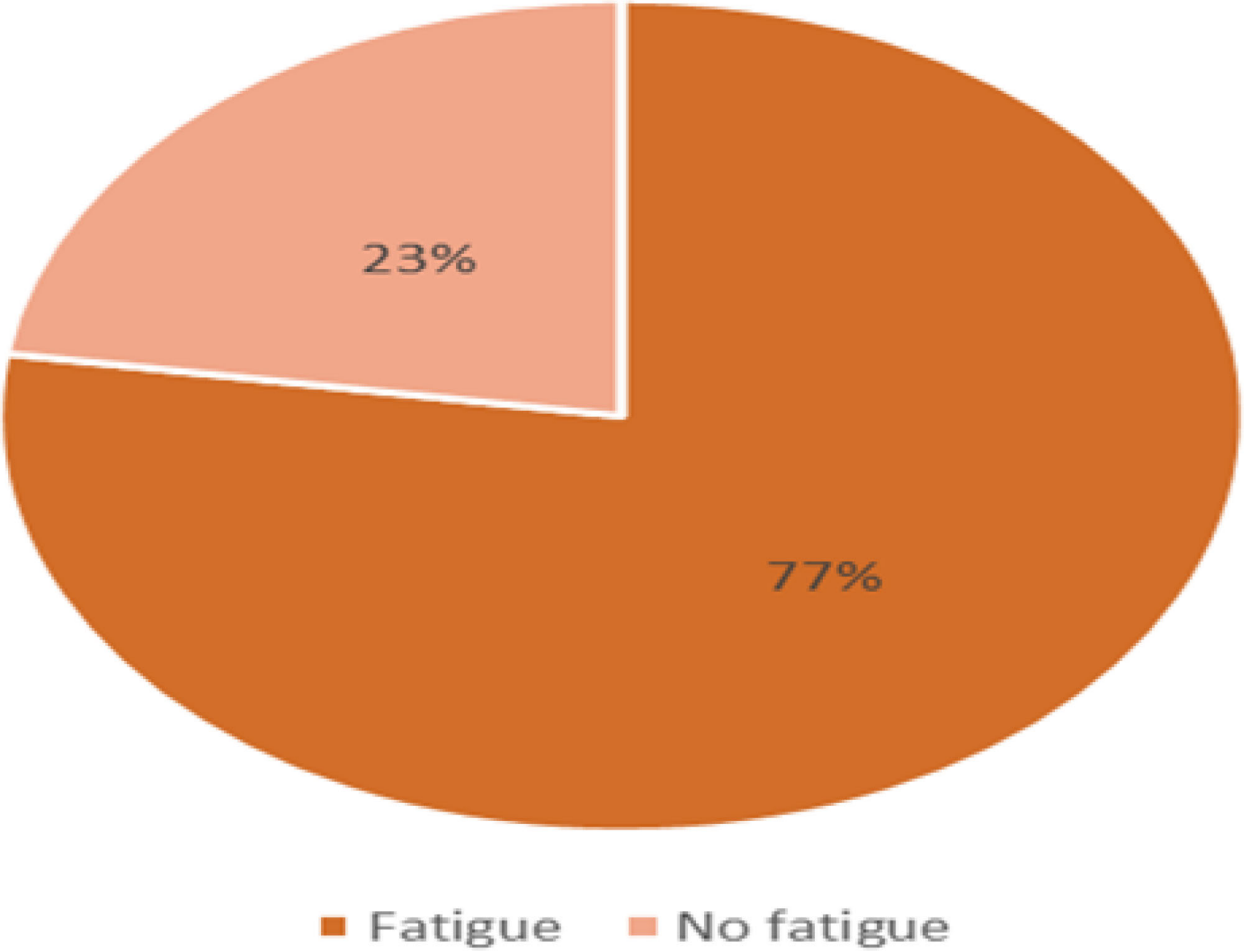
Prevalence of CRF among the study participants at HUCSH, Ethiopia (n = 297).

**Figure 2:**
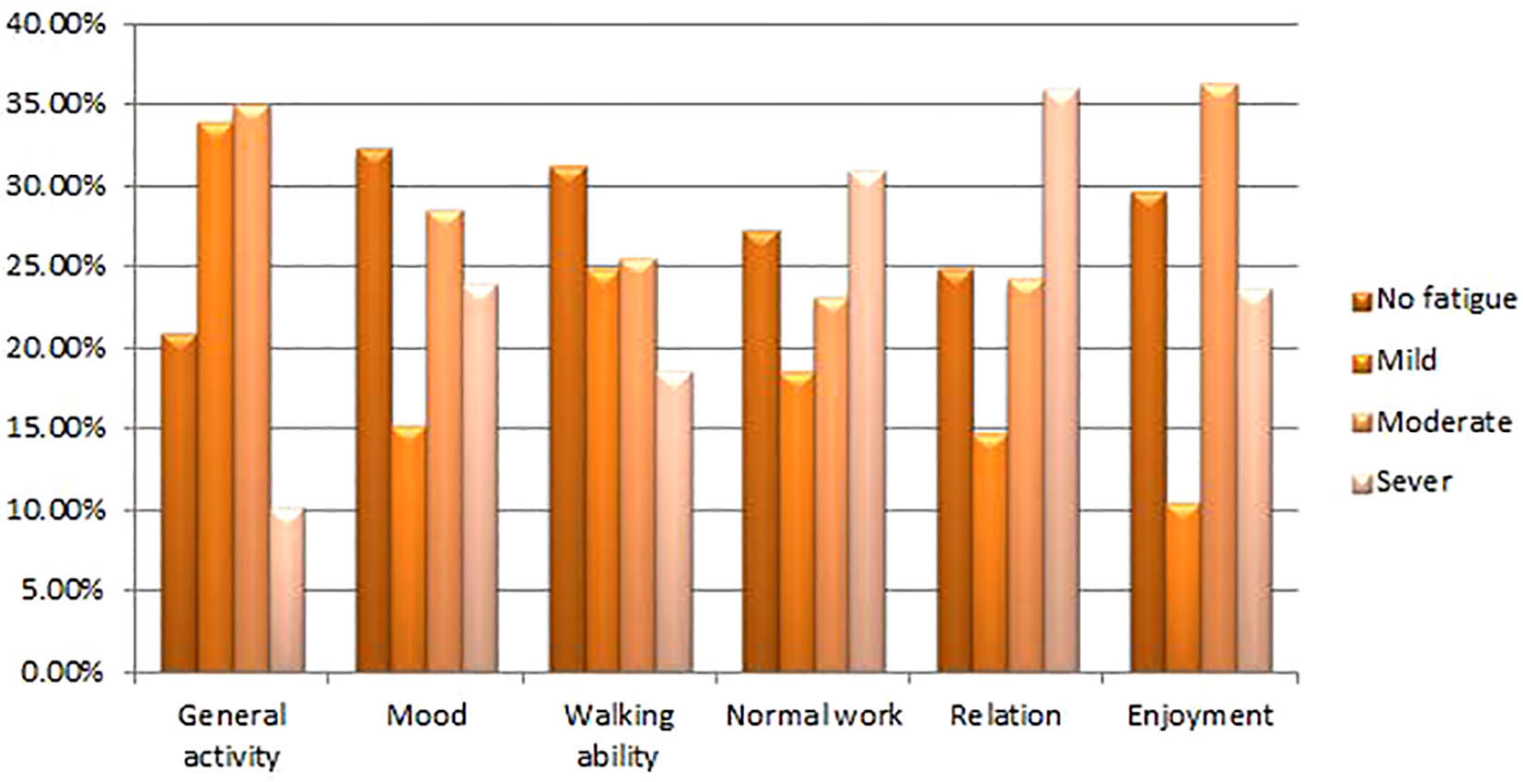
Effect of CRF on the normal activities of the study participants at HUCSH, Ethiopia (n = 297).

The overall fatigue among individuals with cancer in this study was 77.4% (95% CI: 72.7%–82.2%) (**Figure 1**), and the effect of fatigue on daily routine activities is shown below (**Figure 2**).

#### Factors associated with fatigue

Thirteen variables were associated with a bivariate analysis: monthly income, medical expense, type of admission, stage of cancer, presence of infection, disease status, chemotherapy cycles, type of cancer treatment, use of antiemetics, reporting to a physician, anaemic status, comorbid diseases, and type of comorbid diseases. However, only four variables in the multivariate analysis—uninsured medical expense, P value = 0.008, 3.22 (1.37–7.58); late stage of cancer, P value = 0.000, 6.11 (3.06–15.12); anaemic status, P value = 0.009, 3.71 (1.39–9.88); and comorbid diseases, P value = 0.000, 7.22 (4.07–14.84)—were significantly associated with fatigue (**Table 3**).

**Table 3:** Bivariate and multivariate analyses of factors associated with fatigue among the study participants (n = 297).

Variable	Category	Fatigue		COR(95%CI)	AOR(95%CI)	P Value
		No	Yes			
Household income per capital	<500 birr/month	17	86	2.47 (1.17, 5.20)	2.45 (0.73, 8.19)	0.145
	500-1000 birr/month	17	61	1.75 (0.82, 3.74)	2.03 (0.58, 7.08)	0.265
	1100-2000 birr/month	13	42	1.58 (0.69, 3.58)	1.09 (0.29, 4.07)	0.902
	>2000 birr/month	20	41	1	1	1
Medical expense/payments	Insured	40	103	1	1	1
	Uninsured	27	127	1.83 (1.05, 3.18)	3.22 (1.37, 7.58)	0.008*
Type of admission	Out patient	24	179	6.29 (3.49, 11.33)	9.69 (3.49, 26.87)	0.061
	In patient	43	51	1	1	1
Stage of cancer	Early stage	35	49	1	1	1
	Late stage	32	181	4.04 (2.28, 7.17)	6.11 (3.06, 15.12)	0.000*
Presence of infection	Yes	18	120	2.97 (1.63, 5.41)	0.93 (0.34, 2.52)	0.888
	No	49	110	1	1	1
Diseases status	No evidence	20	56	1	1	1
	Local	25	68	0.97 (0.49, 1.93)	0.86 (0.20, 3.65)	0.838
	Metastasis	22	106	1.72 (0.87, 3.42)	0.72 (0.15, 3.43)	0.681
Chemotherapy cycles	First	12	31	1	1	1
	Second	11	27	0.95 (0.36, 2.50)	0.65 (0.14, 3.11)	0.587
	Third	13	25	0.74 (0.29, 1.92)	3.22 (0.59, 17.73)	0.178
	Fourth	16	66	1.59 (0.68, 3.78)	3.23 (0.77, 13.61)	0.110
	5th & above	15	81	2.09 (0.88, 4.96)	1.26 (0.29, 5.44)	0.756
Type of cancer treatment	Chemo & Radiation	14	99	2.26 (1.02, 5.01)	2.77 (0.79, 9.71)	0.111
	Chemotherapy	37	81	0.70 (0.35, 1.39)	0.88 (0.29, 2.63)	0.823
	Surgery	16	50	1	1	1
Use of antiemetic	Yes	50	148	1	1	1
	No	17	82	1.63 (0.88, 3.01)	3.84 (1.26, 11.73)	0.068
Reporting to physician	Yes	41	106	1	1	1
	No	26	124	1.85 (1.06, 3.22)	2.31 (0.91, 5.89)	0.078
Anaemic status	Yes	22	148	0.27 (0.15, 0.48)	3.71 (1.39, 9.88)	0.009*
	No	45	82	1	1	1
Comorbidity diseases	Yes	14	147	6.71 (3.51, 12.81)	7.22 (4.07, 14.84)	0.000*
	No	53	83	1	1	1
Type of comorbidity diseases	Hypertension	2	25	0.85 (0.29, 2.45)	0.50 (0.08, 3.15)	0.461
	HIV/ADIS	2	26	4.17 (0.72, 24.23)	0.37 (0.03, 4.51)	0.434
	DM	0	16	4.33 (0.75, 25.15)	0.97 (0.09, 10.34)	0.979
	Others	5	15	1	1	1

The symbol "*" indicates p < 0.05.

### Qualitative study results

#### Sociodemographic and clinical characteristics of the participants in the qualitative study

The ages of the study participants ranged from 37-70 years, with a mean of 50.0 years (SD ± 9.44). Most participants were males (56.25%), and more than half (56.25%) were from urban areas. With respect to marital status, 62.5% were married, whereas 37.5% were single, divorced, or widowed. In terms of education, 31.25% had no formal education, whereas 68.75% had completed some level of formal education. Concerning employment status before the illness, 37.5% were employed, 43.75% were unable to work because of health problems, and 18.75% were engaged in informal or part-time jobs. The reported income levels were 37.5% who earned <500 birrs per month, 25.0% who earned between 500 and 1000 birrs, and 37.5% who earned >1000 birrs. Fifty percent of the participants had medical insurance.

Clinically, 75.0% of the participants were in advanced stages of cancer (stage III & IV), with anaemia recorded in 62.5% of them. Additionally, comorbid conditions such as hypertension, HIV/AIDS, and diabetes mellitus were recorded in 56.25% of the participants. With respect to the treatment modalities received from the hospital, 50.0% were receiving chemotherapy, 31.25% were receiving combined chemotherapy and radiation, and 18.75% had undergone surgery (**Table 4**).

**Table 4:** Sociodemographic and clinical characteristics of the participants in the qualitative study (n=16).

Variable	Category	Frequency	Percent (%)
Age (years)	Minimum	37	–
	Maximum	70	–
	Mean ± SD	50.0 ± 9.44	
Gender	Male	9	56.25%
	Female	7	43.75%
Residence	Urban	9	56. 25%
	Rural	7	43.75%
Marital status	Married	10	62.5%
	Unmarried	6	37.5%
Education level	No formal education	5	31.25%
	Primary education	4	25.0%
	Secondary education	3	18.75%
	Above secondary education	4	25.0%
Occupation	Employed (before illness)	6	37.5%
	Unemployed/Unable to work	7	43.75%
	Informal/Part-time work	3	18.75%
Household income	< 500 birr/month	6	37.5%
	500–1000 birr/month	4	25.0%
	>1000 birr/month	6	37.5%
Medical insurance	Insured	8	50.0%
	Uninsured	8	50.0%
Cancer stage	Early (Stage I & II)	4	25.0%
	Late (Stage III & IV)	12	75.0%
Anaemia status	Yes	10	62.5%
	No	6	37.5%
Comorbidities	Yes	9	56.25%
	No	7	43.75%
Type of comorbid diseases	Hypertension	3	18.75%
	HIV/AIDS	2	12.5%
	Diabetes Mellitus	2	12.5%
	Other	2	12.5%
Type of cancer treatment	Chemotherapy	8	50.0%
	Chemotherapy & Radiation	5	31.25%
	Surgery	3	18.75%

#### Cancer-related fatigue experiences among the study participants

**Figure 3:**
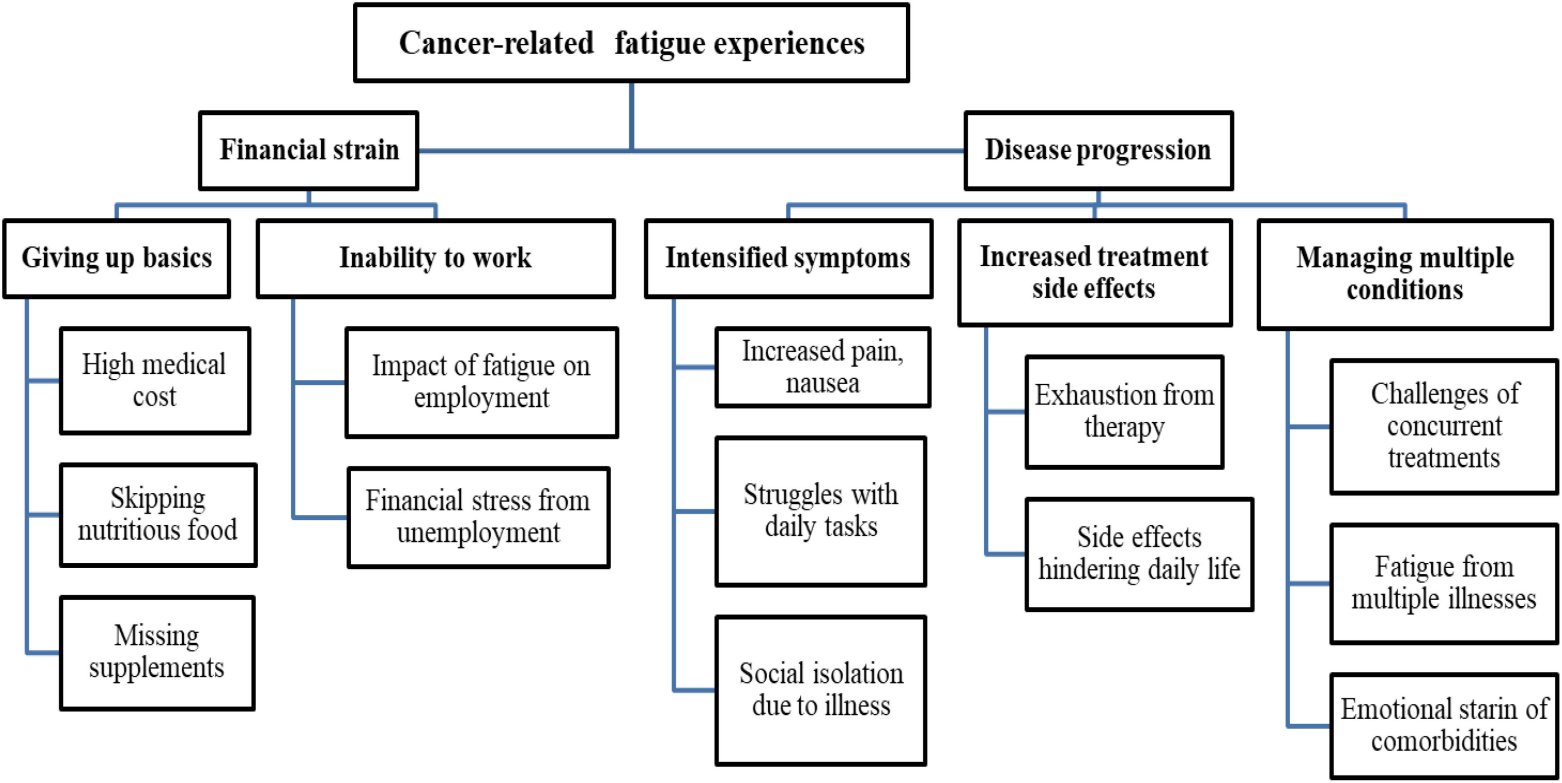
Coding tree for thematic analysis of CRF experiences among the study participants.

Two themes were identified from the participants’ in-depth interviews. Theme I address ‘financial strain’, which was subcategorized into ‘giving up basics’ and ‘inability to work’. Theme II reviews ‘disease progression’, which was subcategorized into ‘intensified symptoms’, ‘increased treatment side effects’, and ‘managing multiple conditions’ (**Figure 3**).

Theme I: Financial strain: Individuals who lack insurance or whose coverage is insufficient typically incur high out-of-pocket costs. This financial strain compels them to make tough choices, such as forgoing necessary medical care or missing prescriptions, which exacerbates their fatigue. Participant 3 shared,

*“Since losing the heavy physical work job, I have been experiencing strained finances that affect my ability to eat nutritious food that could help me get my energy back. This financial stress has put me in such a position that I need to let go of other basic requirements. I have started a small and less demanding job. In trying to manage my energy, I rest throughout the day, hydrate myself by drinking plenty of water, and do some light breathing exercises. That is irritating, because I truly know all those measures are just not enough, but that is the only option left for me.”*

This underlines how a reduced ability to work due to deteriorating health creates a financial burden; hence, managing fatigue and basic needs becomes even harder. Two subthemes underlying the ‘financial strain’ theme are ‘giving up basics’ and ‘inability to work’, both of which emphasize some serious financial strain faced by individuals with cancer.

1. Giving up basics: Individuals complained about the high cost of medical care and supplies, which left them anxious and upset. They also complained about being forced to skip basic necessities such as nutritious food or other medications, which deteriorated their health and energy. This is explained by participant 5, who states “*My main concern is how I will pay for my therapy and prescription medicines. Many times, I have to choose between either paying for treatment or eating healthy. I’m exhausted by the financial strain more than by the cancer itself*.” This is further evidenced by participant 9, who says, “*Each hospital visit comes with additional expenditures that are not covered by insurance. I usually struggle to afford the care I require, and the financial stress simply makes me feel more worn out. I have to skip lunch when going to the hospital to save some money*.” This response underlines not only the financial pressure but also the forced trade-offs between healthcare and basic needs such as food or other medications.
2. Inability to work: Fatigue and declining health make it impossible for a person to work, which increases financial stress and results in a vicious cycle of exhaustion and stress. Participant 2 worried that this inability has been affecting life: “*I am no longer able to work due to fatigue. The financial strain of being unemployed is miserable, and it only adds to my exhaustion*.” This quote emphasizes the double burden of physical fatigue and the emotional and financial consequences of unemployment. Participant 11 further reinforced this view: “*My condition forced me to stop working, and now I struggle to meet my basic demands. This financial hardship heightens my worry and makes me feel even more exhausted*.” Here, the participant describes a similar experience, highlighting how inability to work because of fatigue increases worry and financial hardship. This reflects the broader challenge in managing basic needs while dealing with the dual burden of health issues and financial strain.

Theme II: Disease progression: People experience severe fatigue as a result of the constant occurrence of symptoms that worsen with advanced cancer. Individuals with advanced-stage cancer have less energy and a lower quality of life due to the severe physical consequences of the disease and the intense treatments needed. Additionally, the added stress of managing numerous treatments and symptoms makes fatigue worse for participants who are also managing multiple health conditions. Together, these findings highlight the significant influence that illness progression has on fatigue. Participant 8 explained,

*“Living with progressed cancer is exhausting on every level. All the pains and tiredness are constant, and each day appears to be a battle through the most basic task. On top of that, treatments make me feel that even more worn down chemo makes me so weak that I can hardly move, and the side effects seem to worsen every time. Managing my heart condition along with cancer is another added load because continuous tiredness makes me enter into cycles of exhaustion. It is like my body is fighting a continuous war, and hence I am too tired for anything else.”*

This response captures very well the extent to which this progressive illness burdens individuals physically, emotionally, and mentally while pointing to just how poorer symptoms, treatment side effects, and the management of comorbid conditions add to their overwhelming fatigue. The ‘disease progression’ theme is supported by three subthemes: ‘intensified symptoms’, ‘increased treatment side effects’, and ‘managing multiple conditions’.

1. Intensified symptoms: Participants reported that they continued to experience symptoms such as nausea, pain, and fatigue, which were more severe and frequent as their cancer progressed. Their daily lives are profoundly affected by these symptoms, which makes regular tasks more challenging. Participant 1 shared, “*My illness have become severe and controlling my life. I find it difficult to complete even simple tasks such as getting out of bed or making food because I’m continuously in pain and tired*.” Another participant, participant 4, expressed, “*Nausea and severe weariness have made it impossible for me to attend social occasions. I feel isolated from my friends and family*.” These clarifications reflect that other symptom progression directly contributes to fatigue and impacts the day-to-day life of the participants.
2. Increased treatment side effects: Individuals with advanced-stage cancer require intensive therapies, which results in increased side effects. These adverse effects, such as excessive exhaustion, add to their general level of fatigue. Participant 6 described, “*Chemotherapy for my advanced cancer leaves me exhausted and weak all the time.*” The cumulative effects of multiple treatments were also echoed by Participant 10, who remarked, “*My body is negatively impacted by surgery, radiation and chemotherapy, which leaves me feeling exhausted all the time and unable to perform my daily tasks.*” In severe cases, some individuals felt entirely debilitated by these treatments. Participant 10 stated, “*I am completely exhausted and confined to my bed owing to the extreme side effects of my therapy*.” The intensified side effects of advanced cancer treatments exacerbate overall fatigue, often leaving individuals debilitated and unable to carry out daily activities.
3. Managing multiple conditions: Individuals with extra medical illnesses reported feeling more exhausted as a result of having to deal with a number of different treatment plans and symptoms. The health of these participants has been greatly depleted by the difficulty of managing multiple health conditions at the same time. Participant 7 said, “*Dealing with my heart condition on top of cancer is exhausting. The weariness from managing both is overwhelming*.” Another participant, participant 12, explained, “*Having to juggle treatments for both cancer, and my kidney disease leaves me with no energy at all. It is an endless cycle of fatigue.*” This combination of multiple health issues creates a relentless burden, as Participant 15 reflected: “*Managing diabetes and cancer at the same time makes me feel like my body is constantly fighting. The fatigue is relentless.*” The burden of managing multiple health conditions intensifies fatigue and significantly strains overall well-being.

## Discussion

Fatigue is one of the most distressing symptoms among individuals with cancer, with its magnitude increasing during treatment and persisting even after treatment completion (24). This study revealed that 77.4% (95% CI: 72.7%–82.2%) of the study participants experienced fatigue. This prevalence aligns with studies conducted in the Amhara region (77.3%) (15), Tikur Anbessa Specialized Hospital (TASH) (74.8%) (3), South Africa (80%) (25) and South Korea (79.3%) (26). These consistent rates, even in geographically proximate and socioculturally similar regions, indicate that fatigue is a highly prevalent symptom because of shared physiological and treatment-related factors driving the symptom experience across different populations with cancer. Similar findings from these regions increase the strength of the data and increase the external validity of this study by showing that fatigue remains an issue irrespective of slight regional variation.

The higher prevalence in this study than in similar studies from Italy (62.1%) (27), France (60%) (28), Spain (43%) (29), China (52.07%) (30), and Canada (29%) (31) may be related to several factors that might include dissimilarities in healthcare infrastructures and the availability of supportive care and treatment modalities. In countries with better health infrastructures, individuals may have better access to symptom management and supportive therapies that alleviate fatigue. In addition, the higher prevalence in Ethiopia might further suggest financial burdens, a lack of comprehensive care for cancer, and possible differences in treatment intensity or stage at diagnosis.

Conversely, the lower prevalence of fatigue revealed in this study than the higher rates reported in Iran (89.6%) (32), India (98.30%) (33), Austria (84%) (34), and Taiwan (93.5%) (35) may be attributed to differences in healthcare systems, socioeconomic conditions, disease management practices, and cultural factors that influence both the experience and reporting of fatigue.

One of the significant findings of this study was that participants without medical insurance were three times more likely to experience fatigue than those with insurance. This finding is in agreement with studies from France (36), China (37), and the USA (38). While health-insured individuals are more likely to access early detection and treatment services that could reduce fatigue, uninsured individuals often avoid seeking medical treatment as a result of financial burdens created by costly out-of-pocket expenses, which further exacerbates their condition of fatigue (12, 39). This was further supported by the qualitative findings, wherein participants frequently described having to make difficult choices between healthcare and basic needs, such as food, owing to high out-of-pocket expenses. This financial strain not only limited their ability to access necessary treatments but also heightened their physical and emotional exhaustion. These experiences demonstrate how a lack of insurance exacerbates fatigue by creating barriers to both healthcare and daily living essentials, reinforcing the link between financial hardship and increased fatigue.

The study also established that individuals in the late stages of cancer were six times more likely to experience fatigue than those in the early stages. This agreed with studies from Tikur Anbessa Specialized Hospital (3), Iran (40), China (41), and the USA (42), indicating that late disease stages contribute to increased fatigue. The qualitative data revealed that individuals in progressed stages of cancer experienced more severe and frequent symptoms, which significantly contributed to their fatigue. The participants reported intense physical exhaustion from managing the compounded effects of worsening symptoms, such as pain, and extreme weakness, alongside the debilitating side effects of aggressive treatments such as chemotherapy. Many described the overwhelming burden of trying to cope with multiple health conditions and complex treatment regimens, leading to relentless fatigue. These insights align with the quantitative finding that individuals in the late stage of cancer are far more likely to suffer from profound fatigue than are those in the early stages, as the physical and emotional tolls intensify with disease progression (43, 44).

Participants with anaemia are three times more likely to develop fatigue than those without anaemia, a finding that is consistent with studies from India (45) and the USA (46). Anaemia increases the risk of fatigue in individuals with cancer, especially after cancer treatment (47, 48), as most cancer treatments target the bone marrow, reducing blood counts or red blood cell levels. The reduced oxygen-carrying capacity of the blood (haemoglobin) leads to tissue hypoxia and fatigue (49, 50). The high prevalence of anaemia in this study further highlights the importance of routine blood monitoring and management in reducing fatigue, especially in resource-limited settings where anaemia may be more common owing to nutritional deficiencies or limited access to medical interventions. This is further supported by the qualitative findings of giving up basics, which include food and nutritional support.

Moreover, managing multiple health conditions significantly exacerbates fatigue, with individuals with comorbidities being seven times more likely to develop fatigue than those without comorbidities. This finding contrasts with studies from the Netherlands (51) and Norway (52) but underscores the cumulative impact of multiple health issues on fatigue (53). Qualitative data have shown that managing multiple treatments for different conditions, along with cancer therapies, severely depletes individuals’ energy levels, making fatigue more persistent and pervasive. The participants reported that dealing with several chronic conditions, in addition to cancer, created a constant state of physical and emotional strain. This highlights the need for integrated care approaches that consider both cancer-related fatigue and the challenges posed by comorbidities.

## Limitations

While this study provides valuable findings, especially from its mixed-methods approach, some limitations need to be considered. The cross-sectional design limits the ability to track changes in cancer-related fatigue over time, providing only a single snapshot of data at one point. Self-reporting may result in biased data, as participants might distort their fatigue levels influenced by personal or cultural factors. Additionally, the inclusion of patients with various cancer types, stages, and treatments may have influenced fatigue experiences, limit the transferability of the results to other cancer populations. Furthermore, potential confounding factors, such as cognitive impairments related to CRF, were not specifically addressed. Despite these limitations, the integration of quantitative data with in-depth qualitative insights strengthens the overall contribution of this study in understanding CRF.

## Conclusion and recommendations

This study underscores the high prevalence and severe impact of fatigue among individuals with cancer, with 77.4% of participants reporting significant fatigue. These findings reveal that fatigue is closely linked to several key factors: lack of medical insurance, late-stage cancer, anaemia, and comorbid illness. Individuals without insurance experience heightened fatigue due to financial strain, which exacerbates their condition and limits access to necessary medical care. The late stages of cancer and anaemia contribute to increased fatigue owing to more severe symptoms and side effects from intensive treatments. Additionally, managing multiple health conditions concurrently intensifies fatigue, highlighting the compound effects of chronic illnesses on well-being.

Addressing these factors through early intervention, financial support, and integrated care approaches can help mitigate the impact of fatigue and improve the quality of life for individuals with cancer. Healthcare providers should prioritize tailored strategies to manage fatigue effectively, considering the multifaceted nature of this distressing symptom and its significant implications for care. Future research should focus on multicentre and longitudinal studies to improve generalizability and track fatigue progression over time.

## Data Availability

The original contributions presented in the study are included in the article/supplementary material. Further inquiries can be directed to the corresponding author.
